# Obesity and Aerobic Fitness among Urban Public School Students in Elementary, Middle, and High School

**DOI:** 10.1371/journal.pone.0138175

**Published:** 2015-09-17

**Authors:** B. Ruth Clark, M. Leanne White, Nathaniel K. Royer, Tamara L. Burlis, Nicholas C. DuPont, Michael Wallendorf, Susan B. Racette

**Affiliations:** 1 Program in Physical Therapy, Washington University School of Medicine in St. Louis, St. Louis, Missouri, United States of America; 2 Saint Louis Public Schools, St. Louis, Missouri, United States of America; 3 Division of Biostatistics, Washington University School of Medicine in St. Louis, St. Louis, Missouri, United States of America; National Institute of Health, ITALY

## Abstract

**Aims and Objectives:**

To assess the prevalence of cardiovascular disease risk among urban public school students through a collaborative school district and university partnership.

**Methods:**

Children and adolescents in grades K-12 from 24 urban public schools participated in measurements of height, weight, and other health metrics during the 2009–2010 school year. Body mass index (BMI) percentiles and z-scores were computed for 4673 students. President’s Challenge 1-mile endurance run was completed by 1075 students ages 9–19 years. Maximal oxygen consumption (⩒O_2_max) was predicted using an age-, sex-, and BMI-specific formula to determine health-related fitness. Resting blood pressure (BP) was assessed in 1467 students. Regression analyses were used to compare BMI z-scores, fitness, and age- and sex-specific BP percentiles across grade levels. Chi-square tests were used to explore the effect of sex and grade-level on health-related outcomes.

**Results:**

Based on BMI, 19.8% were categorized as overweight and 24.4% were obese. Included in the obese category were 454 students (9.7% of sample) classified with severe obesity. Using FITNESSGRAM criteria, 50.2% of students did not achieve the *Healthy Fitness Zone* (HFZ); the proportion of students in the *Needs Improvement* categories increased from elementary to middle school to high school. Male students demonstrated higher fitness than female students, with 61.4% of boys and only 35.4% of girls meeting HFZ standards. Elevated BP was observed among 24% of 1467 students assessed. Systolic and diastolic BP z-scores revealed low correlation with BMI z-scores.

**Conclusions:**

A community-university collaboration identified obesity, severe obesity, overweight, and low aerobic fitness to be common risk factors among urban public school students.

## Introduction

Cardiovascular disease risk among children and adolescents is a serious health concern. The national prevalence of obesity among youth ages 6–19 years is estimated to be 18.2% [1]. The prevalence of severe obesity in children and adolescents is increasing and contributes to serious cardiovascular and metabolic health risks [2, 3]. Physical inactivity is another major health concern [4]. Nationwide, only 54% of youth ages 6–19 years achieve the amount of moderate- and vigorous-intensity physical activity recommended in the Physical Activity Guidelines for Americans [5, 6]. The National Center for Health Statistics reports inadequate levels of cardiorespiratory fitness among 57.8% of adolescents ages 12–15 years [7].

Schools serve as an important setting to promote health and wellness and to conduct health-related screenings. Physical education (PE) plays a pivotal role in assessment of fitness. The components of the fitness evaluation and the required grade levels for fitness testing vary according to state mandates. At the time of this study and the writing of this article, Missouri Department of Elementary and Secondary Education (MODESE) required fitness testing and reporting for fifth and ninth grade students [8]. The components of the fitness evaluation include aerobic capacity, upper body strength/endurance, abdominal strength/endurance, and flexibility. Missouri’s benchmarks for passing are based on a combination of performance standards from the President’s Challenge and health-related FITNESSGRAM criteria, as delineated in the MODESE physical fitness manual [8]. Measurement of body composition (e.g., body mass index) was/is optional.

The President’s Challenge 1-mile run is an assessment tool to determine performance-based fitness using age- and sex-specific national standards [9]. FITNESSGRAM criterion-referenced standards were established to provide health-related fitness criteria [10]. FITNESSGRAM became available nationwide during the1985-86 school year and has been adopted by many states as the assessment tool for evaluating fitness in school-aged youth. The President’s Council on Physical Fitness, Sports & Nutrition, the Society of Health and Physical Educators, and The Cooper Institute has adopted the FITNESSGRAM software and assessment tool for determining students’ health-related fitness [10, 11].

We report on an initiative to screen for cardiovascular disease risk factors among urban school-aged youth in partnership with the city’s public school district. The screening assessments were conducted in collaboration with the school district’s Health and Wellness Committee. This project demonstrates a productive and beneficial partnership between a large urban public school district and a research institution. The results of this screening initiative enabled the school district to procure federal and foundation grant funding to implement programs designed to enhance student physical activity, fitness, and health.

The primary aim of this study was to determine the prevalence of obesity, overweight, and low levels of aerobic fitness among urban public school students at all grade levels. A secondary aim was to determine the feasibility of blood pressure screening in a large public school district and to estimate the prevalence of students who need follow-up.

## Materials and Method

An observational, cross-sectional study was conducted to determine the prevalence of cardiovascular disease risk factors among youth attending urban public schools in St. Louis, Missouri during the 2009–2010 school year. Data were analyzed in 2011–2015.

### Participants

The study population comprised children and adolescents in grades K-12 attending 24 of the Saint Louis Public School District’s 75 schools. Overall district enrollment in 2009–2010 was 25,046 students, with racial distribution 80.6% Black, 13.7% White, 2.9% Hispanic, and 2.8% other [12]. Study participants included: (1) students whom we assessed and who provided verbal assent and parent/guardian written permission and (2) students whose de-identified data were extracted from 2009–2010 physical education reports. Date of birth (or age), sex, and measurements of height and weight were required for inclusion in the study population. This study was approved by The Washington University in St. Louis Institutional Review Board and the Saint Louis Public Schools Research Review Committee.

### Measures

#### Anthropometric

Assessments were conducted by members of our research team and by certified Physical Education teachers during PE classes. Height was measured to the nearest ⅛ inch and weight to the nearest 0.1 pound for computation of body mass index (BMI). Centers for Disease Control and Prevention (CDC) SAS code was used to compute BMI-for-age percentiles and BMI z-scores [13]. Weight status was classified according to CDC BMI-for-age criteria: underweight (< 5^th^ percentile), normal weight (5^th^ to < 85^th^ percentile), overweight (85^th^ to < 95^th^ percentile), or obese (≥ 95^th^ percentile) [14]. Severe obesity was defined as BMI ≥ 120% of the 95^th^ percentile of BMI-for-age or BMI ≥ 35.0 kg/m2 [2]. Severe obesity was further classified as class 2 obesity (BMI = 120% to < 140% of the 95^th^ percentile of BMI-for-age or BMI ≥ 35.0 kg/m^2^) or class 3 obesity (BMI ≥ 140% of the 95^th^ percentile or ≥ 40.0 kg/m^2^) [3].

#### Aerobic fitness

Physical education teachers conducted aerobic fitness assessments as a component of the PE curriculum. Maximal oxygen consumption (⩒O_2_max) was predicted from the 1-mile endurance run time using the following age-, sex-, and BMI-specific equation:

⩒O_2_max (ml•kg^-1^•min^-1^) = (.21 × (age × sexcode))—(.84 × BMI)—(8.41 × Mile Time) + (.34 × Mile Time × Mile Time) + (108.94), where age is in years, sexcode is 0 for females and 1 for males, BMI is in kg/m^2^, and mile time is in minutes [15]. Individual estimates of ⩒O_2_max were calculated for students ages 9–19 years in grades 4–12 who completed the 1-mile run in ≤ 13.0 minutes. Estimates of ⩒O_2_max were used to classify students into one of three categories according to FITNESSGRAM age- and sex-specific aerobic capacity criterion-reference standards [10]: *Healthy Fitness Zone (HFZ)*, *Needs Improvement-Some Risk*, or *Needs Improvement-High Risk*. Students with 1-mile run times > 13.0 minutes were included in the *Needs Improvement-High Risk* category. Presidential Youth Fitness Program percentiles (5 percentile increments) were computed for each student using sex- and age-specific national standards [9]. Results are categorized as ≥ 85^th^ percentile (highest fitness category), ≥ 50^th^ to < 85^th^ percentile, and < 50^th^ percentile, in agreement with the Presidential benchmarks for award criteria. An additional category, < 5^th^ percentile, was used to identify students with the lowest fitness.

#### Participation in physical activity

Participation in physical activity was evaluated with the following survey question from the Youth Risk Behavior Surveillance System [16]. “During the past 7 days, on how many days were you physically active for a total of **at least 60 minutes per day**? (Add up all the time you spent in any kind of physical activity that increased your heart rate and made you breathe hard some of the time.)”. The assessment question was administered by paper and pencil to youth age 11–19 years who were attending physical education class.

#### Blood pressure (BP) and pulse rate

Resting BP and pulse rate were assessed twice (with one minute re-filling period) in the right arm at the level of the heart. Assessments were conducted following 5 minutes of quiet seated rest using Omron HEM 907XL IntelliSense Professional Digital Blood Pressure Monitors and appropriate sized cuffs. When errors or disparate readings occurred, BP assessment was repeated by auscultation (sphygmomanometer and stethoscope). Systolic and diastolic BP percentiles were computed from the average of blood pressure recordings using a sex-, age-, and height percentile-specific algorithm with HealthWatch Pro software (version 3.1) to categorize BP status as normal (systolic and diastolic BP < 90^th^ percentile), pre-hypertension (systolic or diastolic BP 90^th^ to < 95^th^ percentile or BP ≥ 120/80 mm Hg), hypertension (systolic or diastolic BP 95^th^ to < 99^th^ percentile), or severe hypertension (systolic OR diastolic BP ≥ 99^th^ percentile) [17]. Pulse pressure (mm Hg) was computed as the systolic BP minus diastolic BP. Mean arterial pressure (mm Hg) was computed as the sum of diastolic BP and 1/3 pulse pressure. Assessments were conducted at each school in a quiet environment and confidentiality was maintained. The procedure was carefully explained to students prior to assessment.

### Data Analyses

Inclusion in the final data set required date of birth (or age), sex, and measured height and weight for the computation of BMI-for-age percentile as the primary outcome. Analyses of BMI-for-age percentiles and BMI z-scores were performed using Statistical Analysis Software (SAS Institute, Cary, NC, version 9.1, 9.3); biologically implausible values (BMI z-score < -4 or > +5) were removed from the final data set. Fitness analysis excluded students for whom a distance of 1 mile was not specified on the fitness form. Grade level for each student was coded as elementary (grades K-5), middle school (grades 6–8), or high school (grades 9–12). Cross-tabulated frequency tables were generated for dichotomous and unordered categorical variables; summary statistics were generated for continuous and ordered categorical variables. BMI-for-age percentiles and BMI z-scores across grade levels are reported as means (SD) with 95% confidence intervals of the mean. Categorical variables (i.e. weight status and healthy fitness zone) are reported as percent of the sample. BP percentiles are reported as median values with bootstrap 95% confidence intervals. General linear models were used to explore the relationship between BMI z-score and performance-level fitness and to compare BP percentiles between grade levels. Chi-square tests were used to explore sex and grade-level effects on weight status (dichotomized as underweight/normal weight and overweight/obese), fitness (dichotomized into *HFZ* and not-*FHZ)*, and BP status. Pearson correlation coefficients were computed to explore associations between BMI z-scores and BP z-scores. A *p* value < .05 was considered significant.

## Results

A total of 4894 student records were examined for inclusion in the final data analysis; 221 records were eliminated for incomplete or implausible data, resulting in a final sample of 4673 students in grades K-12 from 8 elementary, 10 middle, and 6 high schools. Mean age was 13.0 years, with a range of 4.7–19.9 years; 46% of students were female and 54% male. Racial / ethnic distribution was 71.4% Black, 15.7% White, 8.8% Hispanic, and 4.1% other for the 2866 students whose race / ethnicity was reported. Aerobic fitness results were available for a subsample of 1075 students, BP results for a subsample of 1476 students, and participation in physical activity for 1398 students.

### Body Mass Index and Weight Status

BMI and weight status were determined for 4673 youth. Weight status results, determined by sex-specific BMI-for-age criteria, revealed that 2.1% of the student sample was underweight, 53.7% normal weight, 19.8% overweight, and 24.4% obese. Age and anthropometric characteristics by grade level are shown in Table 1. BMI-for-age percentiles and BMI z-scores were not statistically different across grade levels.

**Table 1:** Body Mass Index and Weight Status of Urban School Students by Grade Level.

**Variables**	**Elementary (n = 1256)**		**Middle School (n = 2292)**		**High School (n = 1126)**	
	**Mean (SD)**	**95% CI**	**Mean (SD)**	**95% CI**	**Mean (SD)**	**95% CI**
Age, years	9.7 (1.8)	[9.6, 9.8]	13.3 (1.0)	[13.3, 13.4]	16.2 (1.1)	[16.2, 16.3]
BMI, kg/m^2^	20.0 (5.0)	[19.7, 20.2]	23.2 (5.9)	[23.0, 23.5]	24.9 (5.7)	[24.5, 25.2]
BMI-for-age-percentile	68.5 (31.0)	[66.8, 70.2]	71.7 (27.0)	[70.6, 72.8]	70.9 (26.9)	[69.3, 72.4]
BMI z-score	0.72 (1.24)	[0.65, 0.79]	0.83 (1.06)	[0.78, 0.87]	0.77 (1.03)	[0.71, 0.83]
^a^ **Weight Status**	**Elementary, % of population**		**Middle School, % of population**		**High School, % of population**	
Underweight (< 5^th^ percentile)	4.1		1.4		1.3	
Normal weight (5^th^ to < 85^th^	50.5		54.1		56.2	
Overweight (85^th^ to < 95^th^)	19.7		19.5		20.4	
Obese (includes severe obesity, ≥ 95^th^)	25.7		24.9		22.0	
^b^ Severe Obesity (≥ 120% of 95^th^)	9.4		10.4		8.4	

Abbreviations: BMI (Body Mass Index)

^a^ Centers for Disease Control and Prevention categories based on BMI-for-age percentile [14].

^b^ Severe obesity is defined as BMI ≥ 120% of 95^th^ percentile of BMI-for-age or BMI ≥ 35.0 kg/m^2^ [2].

**Fig 1:**
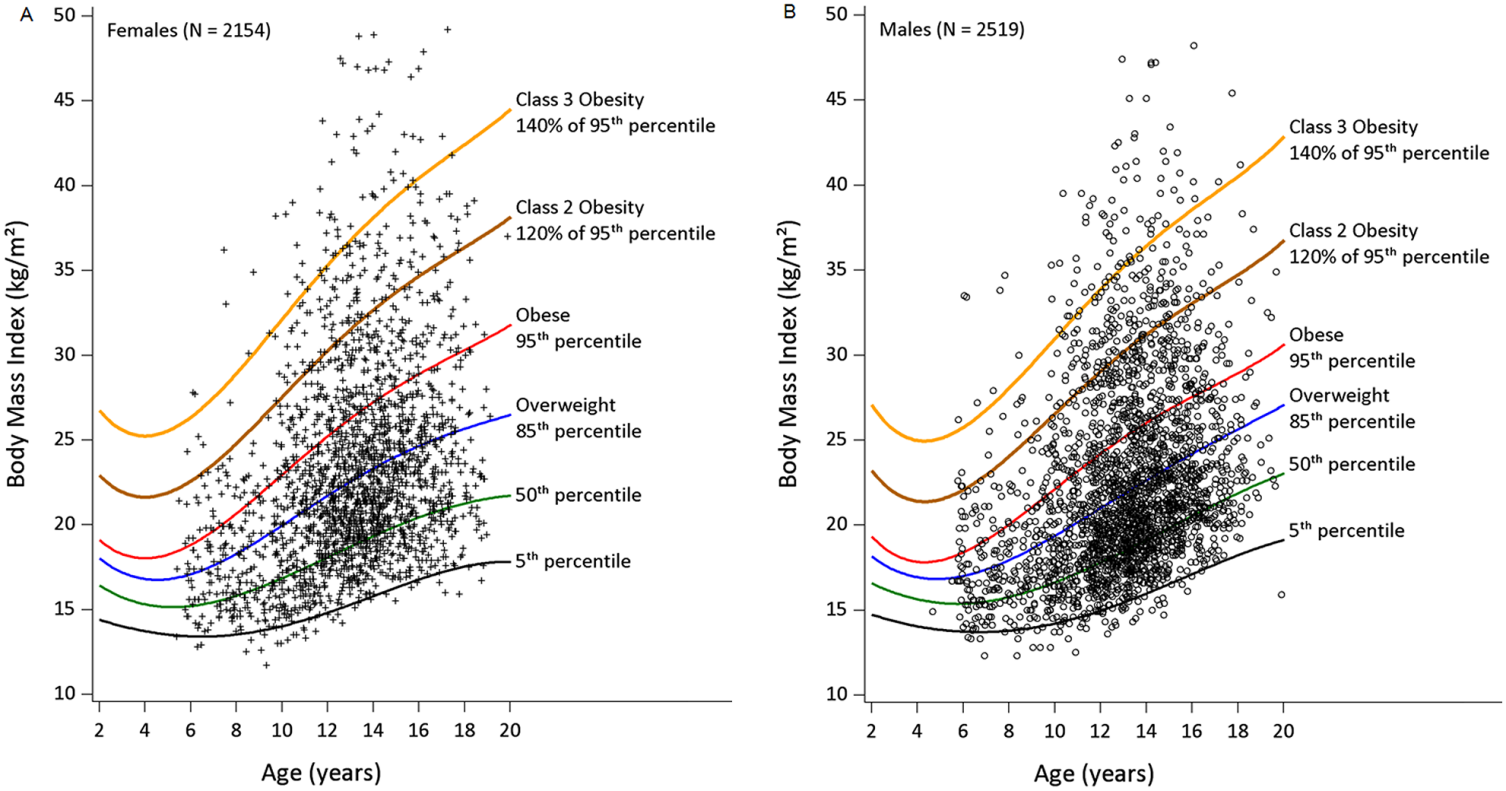
Body Mass Index and BMI-for-age Percentiles of Urban Female and Male Students. Body mass index (BMI) for female students (A) and male students (B) depicted on BMI-for-age percentile growth charts [14]. Each symbol represents one student’s BMI (N = 4673). Severe obesity was defined as class 2 obesity (≥ 120% of the 95^th^ percentile of BMI-for-age) or class 3 obesity (≥ 140% of the 95^th^ percentile) [2].

Fig 1 shows individual student BMI values plotted on BMI-for-age percentile growth charts for 2154 female students (A) and 2519 male students (B). Severe obesity was present in 9.7% of the sample and is depicted with curves indicating class 2 and class 3 obesity. Weight status dichotomized as underweight/normal weight versus overweight/obese showed no statistically significant effect by grade level, *X* ^*2*^ (2, *N* = 4671) = 4.13, *p* = 0.13, or by sex, *X* ^*2*^ (1, *N* = 4671) = 2.61, *p* = 0.11. The classification of severe obesity showed no statistical difference between female students and male students, *X* ^*2*^ (1, *N* = 4671) = 0.28, *p* = 0.60.

### Aerobic Fitness

One-mile aerobic fitness results by grade level are shown in Table 2. The range of run times was 5.2–26.8 minutes, with a mean run-time of 11.6 (SD 3.6) minutes. The proportion of students who achieved the FITNESSGRAM *Healthy Fitness Zone* (HFZ) for aerobic capacity was 49.8%. Male students demonstrated higher fitness than female students, *X* ^*2*^ (1, *N* = 1074) = 74.96, *p* < .001, with 61.4% of male students and 35.4% of female students meeting the HFZ category. The proportion of students in the HFZ decreased from elementary to middle school to high school, *X* ^*2*^ (2, *N* = 1074) = 48.70, *p* < 0.001.

**Table 2:** Aerobic Fitness and FITNESSGRAM Classification of Urban School Students by Grade Level, Age ≥ 9.0 years.

Variables	All Students Grades 4–12	Elementary School Grades 4–5	Middle School Grades 6–8	High School Grades 9–12
N	1075	155	656	264
^a^1-Mile run time, minutes	11.6 (3.6)	11.0 (2.1)	11.4 (3.8)	12.1 (3.9)
^b^ ⩒O_2_max, ml•kg^-1^•min^-1^	43.6 (6.2)	43.1 (4.3)	43.9 (6.6)	43.3 (6.2)
^c^Healthy Fitness Zone	49.8%	67.5%	51.8%	34.5%
^c^Needs Improvement-Some Risk	10.8%	10.4%	8.7%	16.3%
^c^Needs Improvement-High Risk	39.4%	22.1%	39.5%	49.2%

^a^1-mile run time and ⩒O_2_max are reported as mean (SD).

^b^ *n* = 766 for ⩒O_2_max because maximal oxygen consumption cannot be calculated for 1-mile run times > 13.0 minutes.

^c^ FITNESSGRAM criterion-referenced fitness standards [10], reported as % of population for each grade level designation. Students with 1-mile run times > 13.0 minutes were included in the *Needs Improvement-High Risk* category.

**Fig 2:**
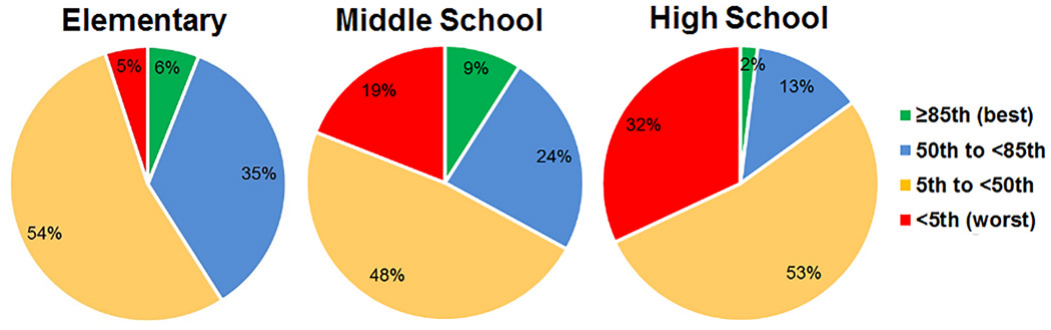
Aerobic Fitness Results from The President’s Challenge 1-mile Endurance Run. Performance level national standards were used to classify fitness of urban school students. Percentage of the population at each grade level (elementary, middle, and high school) is shown. The 85^th^ and 50^th^ percentiles are benchmarks for Presidential awards.

Fig 2 depicts the proportion of students by grade level in the President’s Challenge fitness categories and an additional category, < 5^th^ percentile (very low fitness). Overall, 69.8% of the sample was below the 50^th^ percentile fitness benchmark. Regression analysis of the relationship between BMI z-score and one-mile run performance revealed no correlation among elementary students and low inverse correlations among middle and high school students.

### Participation in Physical Activity

In a sample of 1398 students in grades 5–12 who completed the physical activity questionnaire, only 15.5% reported participating in 60 minutes of physical activity 7 days per week, while 59.1% reported ≤ 3 days per week.

### Blood Pressure and Pulse Rate

Blood pressure and pulse rate assessments were completed on 1476 students (53% female). The subsample of students whose resting BP and pulse rate was assessed had similar BMI, weight status, and aerobic fitness levels as the overall sample. The mean systolic BP percentile was 51.7 (SD 29.2) and the mean diastolic BP percentile was 51.5 (SD 24.5). Table 3 shows summary statistics by grade level for BP indices and resting pulse rate. The majority of students (76.3%) had normal BP; elevated BP was observed in 23.7% of the sample (13.6% pre-hypertension, 6.8%, hypertension, and 3.3% severe hypertension). There was a statistically significant effect of sex, *X* ^*2*^ (3, *N* = 1467) = 53.1, *p* < 0.001. The prevalence of elevated BP was 31.8% among male students and 16.5% in female students. Low correlations were observed between BMI z-score and systolic BP z-score (r = .33, *p* < .001) and between BMI z-score and diastolic BP z-score (r = .30, *p* < .001). Pulse pressures widened across grade levels and mean arterial pressure increased from elementary to middle to high school.

**Table 3:** Resting Blood Pressure Indices and Pulse Rate of Urban School Students by Grade Level.

Variables	Elementary School Grades 2–5 (*n* = 258)		Middle School Grades 6–8 (*n* = 870)		High School Grades 9–12 (*n* = 339)	
	Median	95% CI	Median	95% CI	Median	95% CI
Systolic BP Percentile	38	[34, 46]	57	[53, 61]	53	[50, 57]
Diastolic BP Percentile	46	[39, 53]	53	[50, 56]	50	[46, 53]
	**Mean**	**(SD)**	**Mean**	**(SD)**	**Mean**	**(SD)**
Pulse pressure, mm Hg	39	(9)	46	(10)	48	(11)
Mean Arterial Pressure, mm Hg	75	(9)	80	(9)	82	(9)
Resting pulse rate, bpm	85	(11)	81	(13)	78	(12)

BP percentiles were computed using sex-, age-, and height percentile-specific criteria [17] and data are reported as medians with bootstrap 95% confidence intervals of the median.

Pulse pressure, mean arterial pressure, and resting pulse rate are reported as mean (SD).

## Discussion

Multiple modifiable cardiovascular disease risk factors were observed among urban public school students in St. Louis, MO, including obesity, low levels of aerobic fitness, and low participation in physical activity. The combined prevalence of obesity and overweight among youth during the 2009–2010 school year was 44.2%. A particular concern is the presence of severe obesity in 9.7% of our study population. Furthermore, a large percentage (50.2%) of students did not meet the *Healthy Fitness Zone* standard, with 39.4% classified in the *Needs Improvement-High Risk* category, indicating current or future health risk. Another concerning observation was the decline across grade levels in the percentage of students meeting fitness standards. The FITNESSGRAM category *Healthy Fitness Zone* designates a level of fitness required for low risk for future health problems [10, 18].

The prevalence of overweight and obesity in our urban student sample was higher than the national prevalence of 33.2% reported for the 2009–2010 National Health and Nutrition Examination Survey (NHANES) sample of youth ages 6–19 years [1]. Likewise, severe obesity in our student population was higher than prevalence estimates of 4.0–6.0% [2] and 8.0% [3]. When compared with other urban samples, the prevalence of obesity in our students in 2009–2010 (24.4%) was higher than that observed among youth in grades K-12 in Philadelphia in 2009–2010 (20.5%) [19] and among youth in grades K-8 in New York City in 2010–2011 (20.7%) [20]. One explanation for the high prevalence of overweight and obesity in our sample may be the high proportion of racial minorities (71.4% Black). In comparison, the Philadelphia sample was 59.3% Black, 14.2% White, and 16.7% Hispanic and the corresponding distribution in New York City was 28.4%, 15.2%, and 40.3%, respectively.

Physical fitness plays a key role in promoting health and reducing disease risk. Evaluation of NHANES data (1999–2002) of adolescents aged 12–19 years demonstrated that higher levels of cardiorespiratory fitness were associated with better insulin sensitivity (i.e., less insulin resistance), especially in male students [21]. For all youth, elevated BMI and waist circumference (reflecting abdominal obesity) were predictors of insulin resistance [21]. Additional analyses of NHANES data have shown that adolescents with low fitness have higher BMI, higher waist circumference, higher systolic BP, and higher total cholesterol levels compared to age-matched youth with moderate or high fitness levels [22].

At the time of our assessments, no state required schools to conduct BP screening. Our collaboration with school personnel demonstrated that in-school screening of BP was practical and revealed a need for follow-up assessments. Youth in middle school and high school grades in this sample exhibited higher median systolic BP percentiles compared to the national reference group [17], whereas elementary-aged children in our sample were below the 50^th^ percentile for both systolic and diastolic BP. The prevalence of elevated BP among youth is increasing nationwide [23, 24]. Overweight and obesity are recognized as major contributors to elevated BP in pediatric patient populations [25]. Children and adolescents with prehypertension are at increased risk for left ventricular hypertrophy [26] and for persistence of hypertension into adulthood. The risk for elevated BP is increased in youth ≥ 10 years of age with BMI-for-age percentile ≥ 85^th^ [27]. A longitudinal study with a 21-year follow-up revealed that large pulse pressures measured during adolescence correlated with increased carotid intima-media thickness in adulthood [28]. BP screening with a plan for follow-up, referral, and treatment is essential if the goal is to decrease cardiovascular disease risk among youth [29].

Residents in the city of St. Louis are at higher risk for mortality from diabetes, heart disease, stroke, and cancer compared to residents in the state of Missouri and the USA [30]. Elevated disease risk and reduced life expectancy in specific zip codes in St. Louis are related to neighborhood inequity in the availability of healthful food, neighborhood safety, and access to health care. Relative to Missouri and the U.S., St. Louis has a higher proportion of residents who are Black (49.2% in St. Louis vs. 11.6% in MO and 13.2% in the U.S.) and who live below the poverty level (27.0% vs. 15.0% and 14.9%) [31–33]. Although the prevalence of obesity in children and adolescents was reported to have stabilized nationwide [1], ethnic and socioeconomic disparities in the prevalence of obesity and severe obesity among urban youth are persistent and disturbing [19, 20].

### Study Limitations

Although we assessed a large number of students, with nearly 20% of the student body represented in our BMI measures, the fitness and BP evaluations reflect less than 6% of students enrolled during that school year. Furthermore, BP was assessed on a single occasion for screening purposes, which may have resulted in an overestimate of elevated BP. Many students did not achieve the *Healthy Fitness Zone* based on their 1-mile run time; however, we cannot determine whether their performance was due to low fitness, lack of motivation, or a combination of factors. Finally, elementary schools in this district vary with respect to the grades they offer (e.g., K-5, K-6, K-8); therefore, some students whose grade level was coded as middle school (grades 6–8) were in an elementary school environment. The influence of race could not be determined because our sample was predominantly Black.

## Conclusion

Urban public school children in St. Louis have a high risk for obesity, overweight, and low aerobic fitness. Widespread screening initiatives can be implemented in the school setting to identify important modifiable risk factors among youth. Our study highlights the magnitude of disease risk and the importance of screening students for obesity and severe obesity. Importantly, aerobic fitness assessments using health-related criteria and performance-based standards may best identify youth with low levels of physical fitness. We also demonstrated the feasibility of blood pressure screening in urban public schools. Community-university collaborations enhance opportunities for external funding to support curricular development and new programs that benefit all students. An outcome of our collaborative initiatives have included major federal and foundation funding awards to support an enhanced physical education curriculum, physical activity initiatives, and nutrition education throughout the school district.
